# Could the Age Difference of a Single Calendar Year between Patients Undergoing IVF at 34, 35 or at 36 Years Old Affect the IVF Outcome? A Retrospective Data Analysis

**DOI:** 10.3390/medicina56020092

**Published:** 2020-02-24

**Authors:** Konstantinos Pantos, Konstantinos Sfakianoudis, Sokratis Grigoriadis, Evangelos Maziotis, Petroula Tsioulou, Anna Rapani, Polina Giannelou, Anastasios Atzampos, Sevasti Koulouraki, Michael Koutsilieris, Nikolaos Vlahos, George Mastorakos, Mara Simopoulou

**Affiliations:** 1 Centre for Human Reproduction, Genesis Athens Clinic, 14–16, Papanikoli, 15232 Athens, Greece; info@pantos.gr (K.P.); sfakianosc@yahoo.gr (K.S.); lina.giannelou@gmail.com (P.G.); 2 Department of Physiology, Medical School, National and Kapodistrian University of Athens, 75, Mikras Asias, 11527 Athens, Greece; sokratis-grigoriadis@hotmail.com (S.G.); vagmaziotis@gmail.com (E.M.); petroulatsi@yahoo.gr (P.T.); rapanianna@gmail.com (A.R.); tasosatz.et@gmail.com (A.A.); mkoutsil@med.uoa.gr (M.K.); 3 Second Department of Obstetrics and Gynecology, Aretaieion Hospital, Medical School, National and Kapodistrian University of Athens, 76, Vasilisis Sofias Avenue, 11528 Athens, Greece; sevasti.koulouraki@gmail.com (S.K.); nfvlahos@gmail.com (N.V.); mastorakg@gmail.com (G.M.)

**Keywords:** assisted reproduction, infertility, in vitro fertilization, maternal age, age 35, IVF success

## Abstract

*Background and Objectives:* Clinicians are called to overcome age-related challenges in decision making during In Vitro Fertilization (IVF) treatment. The aim of this study was to investigate the possible impact of a single calendar year difference among patients aged 34, 35 and 36 on IVF outcomes. *Materials and Methods:* Medical records between 2008 and 2019 were analyzed retrospectively. The study group consisted of women diagnosed with tubal factor infertility. Sample size was divided in three categories at 34, 35 and 36 years of age. Embryo transfer including two blastocysts was performed for every patient. Comparisons were performed regarding hormonal profile, response to stimulation, quality of transferred embryos, positive hCG test and clinical pregnancy rate. *Results:* A total of 706 women were eligible to participate. Two-hundred and forty-eight women were 34, 226 were 35 while the remaining 232 were 36 years old. Regarding the hormonal profile, the number of accumulated oocytes and the quality of embryos transferred, no statistically significant difference was documented between the three age groups. Women aged 34 and 35 years old indicated a significantly increased positive hCG rate in comparison to women aged 36 years old (*p*-value = 0.009, *p*-value = 0.023, respectively). Women aged 34 and 35 years old presented with a higher clinical pregnancy rate in comparison to those aged 36 years old (*p*-value = 0.04, *p*-value = 0.05, respectively). *Conclusion:* A calendar year difference between patients undergoing IVF treatment at 34 or 35 years of age does not appear to exert any influence regarding outcome. When treatment involves patients above the age of 35, then a single calendar year may exert considerable impact on IVF outcome. This observation indicates that age 35 may serve as a valid cut-off point regarding IVF outcome.

## 1. Introduction

Childbearing beyond the age of 35 has experienced an increasing trend in developed countries during the last few decades. A striking example is the United States of America (USA), where during the period between 1970–2000, the number of pregnancies for women over 35 has increased eight-fold [1]. The apposite term employed for women over the age of 35 who experience pregnancy is Advanced Maternal Age (AMA) [1]. The highly demanding aspects of modern life, the rigorous pursuit of educational and professional growth combined with the efforts towards acquiring a high standard of living pose as catalysts resulting in the delay of marriage and the acquisition of offspring [2]. However, as clearly documented, AMA may be related to infertility, thus in this case it may stand as an issue jeopardizing a couple’s efforts to achieve a natural conception. Nonetheless, the abundancy of options offered in the field of assisted reproduction, from oocyte cryopreservation to oocyte donation, paves the way for a ground-breaking era in reproduction [2].

A plethora of published data suggests that AMA exerts a negative impact on pregnancy outcomes accompanied with poor prognosis, both of which are associated with a variety of complications during both the gestational and delivery stage [3,4,5,6,7,8,9,10]. In addition, women over 35 report poorer results during In Vitro Fertilization (IVF) treatment due to inadequate ovarian stimulation as well as a lower embryo implantation rate [11,12]. The aforementioned risks certainly translate as concerns, rendering management of such patients within the assisted reproduction context as a rather challenging task for fertility specialists.

Women over the age of 35 represent a considerable percentage of IVF patients. Regarding management, in light of various risks and complications related to AMA, clinicians are confronted with a series of concerns and ethical considerations, incorporating both risks and advantages in the decision-making process. Concerning oocyte or embryo donation, as well as gestational surrogacy, clinicians are called to set an age cut-off point lacking any conclusive guidelines. Despite guidelines [13,14], empirical decisions often dictate the adopted strategy while the demand for a universal protocol has been voiced [15]. A typical example refers to the maximum number of embryos transferred in a single cycle. According to existing guidelines, the age cut-off point dictating this decision is 35 years, whereas for younger women, elective single embryo transfer (eSET) is preferred [16]. In Europe in particular, this decision normally complies with specific legislation. In Belgium, for women under the age of 36, the number of embryos transferred is limited to one, whereas in France and Sweden the maximum number of embryos is two. In Greece, according to current legislation the cut-off point is the age of 35, while for women under the age of 35 years, transferring two embryos is allowed exclusively under specific circumstances. In Germany, for patients under the age of 37, the maximum number of embryos that can be transferred is three. Finally, in the United Kingdom and the Netherlands, eSET is considered to be the method of choice [17].

The age cut-off points employed by clinicians towards decision making in Assisted Reproduction Technology (ART) vary, despite extensive research dedicated to shedding light to the impact of AMA on fertility [15,18,19]. Indicatively, there are different schools of thought on whether the age of 35 [20,21] or the age of 40 [22] should be adopted as the appropriate cut-off point when associating AMA to a statistically significant negative prognosis and pregnancy outcome.

Taking into account the divergent schools of thought and respective practices, studies investigating the clinical outcome regarding immediate age surrounding the presumed cut-off point are timely and essential. What a difference could a single calendar year make regarding the IVF outcome when we consider a patient’s journey undergoing treatment from 34 to 35 and 36 years of age? Addressing this question is of added value equally from the perspective of the practitioner and the patient. Does deviating by a single year from the cut-off point of 35 present with substantial differentiation regarding decision-making on the number of embryos transferred? What is the real impact of age in trying to minimize complications such as multiple pregnancies and maximize the possibility of a positive outcome? How does exact age weigh in the equation? These issues emphasize the crucial nature of this study. The scope of this study is to address the issue of whether a single calendar year difference between patients undergoing IVF treatment at age 34, 35 and 36, respectively, could affect the IVF outcome. Further to that, results could provide insight with regards to how appropriate it is to consider the age 35 as a cut-off point in IVF treatment for patients showcasing a positive reaction to stimulation. Notably, since the existing literature principally reports on data referring to age cohorts of a wider range, the present study contributes in a unique manner. To the best of our knowledge, this report explores—for the first time—the impact of exact age employing a comparison between the adjacent calendar years 34 and 36 surrounding 35 as the age cut-off point in association to a positive prognosis for IVF.

## 2. Materials and Methods

A clinic’s medical records were investigated from 2008 to 2019 in order to recruit patients for this retrospective data analysis. The Genesis Athens Clinic Hospital Ethics Board approved the study protocol in accordance with the Helsinki declaration (130/26/3/2019). Tubal factor infertility was the filed diagnosis for the patients who were submitted to a single IVF treatment cycle. The implemented inclusion criteria enabled recruitment of women with primary infertility attributed to a tubal factor which was confirmed employing hysterosalpigography indicating tubal blockage or a removed salpinx following hydrosalpinx diagnosis or fallopian tube(s) blockage. This etiology of patients’ infertility was chosen during conceptualization of the study due to its favorable clinical end-point potential. The women included were normo-ovulatory with regularity of menstruation of 24–35 days.

The Controlled Ovarian Hyperstimulation (COH)’s protocol of choice was the standard Gonadotropin-Releasing Hormone (GnRH) long agonist protocol. On the 21st day of the cycle, 0.1 mg GnRH agonist was administered. A daily prescription of gonadotropin at 300 international units (IU) was also administrated. Sonographic assessment of follicular development was used as a guide for the adjustment of gonadotropin dose. Oocyte retrieval procedure was scheduled for 36 h following Human Chorionic Gonadotropin (hCG) injection. Progesterone administration was opted for to provide luteal support. An acquisition of ≥10 oocytes was considered as a good ovarian response.

Three major groups were formulated based on patients’ age, namely 34, 35 and 36 years of age. Embryo transfer procedure was performed on day 5, including two embryos at the blastocyst stage. Patients were further subcategorized based on the criterion of embryo quality during the day 5 Embryo Transfer (ET) procedure. Two top quality embryos at the blastocyst stage graded as 4AA, 5AA, and 6AA were categorized in the group of D5A. Any other embryos at the blastocyst stage were characterized as non-top quality embryos. Category D5B included a top-quality blastocyst along with a non-top quality one. Lastly, category D5C consisted of two blastocysts that were both assessed as non-top quality. Gardner’s blastocyst grading system was employed as the grading system of choice in assessing the blastocyst [23].

Standard IVF was the insemination technique performed for all patients included in the present data analysis. We aimed to exclude any issues that may hinder a positive end-point in cases of participants with good prognosis and consequently pose as confounders jeopardizing this study, meaning male factor infertility or any further etiologies jeopardizing the couple’s fertility potential were excluded. The aforementioned inclusion criteria were meticulously selected to confirm and establish that solely good prognosis patients with enhanced probability to achieve pregnancy following a single IVF treatment would be incorporated in the present analysis. According to their files, patients meeting the inclusion criteria were sorted into the abovementioned age groups.

A statistical analysis was conducted to perform a comparison in regards to the basic hormonal profile between the three groups of patients. Profiling included Follicle Stimulating Hormone’s (FSH)*,* Luteinizing Hormone’s (LH) levels, Estradiol’s levels and Anti-Müllerian Hormone’s (AMH) levels. Measurement of FSH and LH levels was conducted on the 3rd day of the menstrual cycle, while estradiol’s levels were determined on the day of HCG trigger employing chemiluminescent microparticle immunoassay on a Roche Immunoanalyser (Roche Cobas e 411). On the 3rd day of the menstrual cycle, an analysis of AMH levels was requested, employing AMH Gen II chemiluminescent microparticle immunoassay on a Roche Immunoanalyser (Roche Cobas e 411).

A statistical comparison was conducted amongst the three age groups including: The number of oocytes accumulated, the number of normally fertilized oocytes, the assessment of quality of embryos included at embryo transfer, the positive hCG test rate, as well as the clinical pregnancy rate. Assessment of a positive hCG test rate was performed based on the number of times that a positive beta human chorionic gonadotropin test (beta hCG) was detected in maternal serum seven days post blastocyst transfer. Subsequently, assessment of clinical pregnancy rate was performed based on the cases in which ultrasound detection of a fetal heart beat confirmed pregnancy 6–7 weeks following the last menstruation.

The R Programming Language for Statistical Purposes was employed for all data analyses. Profiling of hormonal levels, the number of accumulated oocytes and the number of normally fertilized oocytes were compared amongst the three age groups with One Way ANOVA test and Bonferroni Correction Post-hoc analysis. Contingency chi-squared test was applied to compare the transferred embryos’ quality, the positive hCG test rate and the clinical pregnancy rate. Confidence Intervals (CI) of 95% were calculated for each variable and *p*-value < 0.05 was considered statistically significant.

## 3. Results

**Figure 1:**
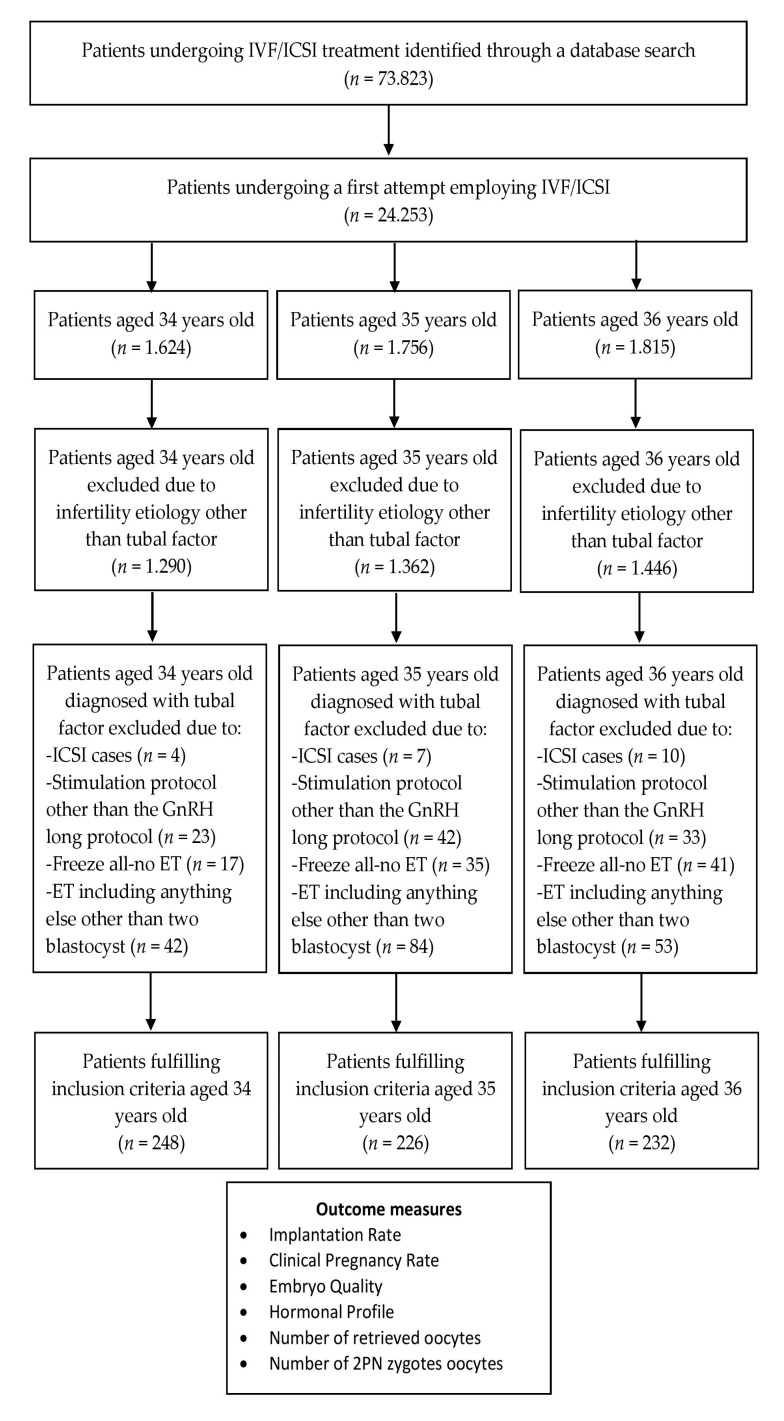
Flow chart on the study group selection process performed.

A total of 706 women were eligible to be included in the current study. Women were categorized to the respective age group based on their age during their first attempt. Thus, two-hundred and forty-eight women were included in the 34 years old group, 226 in the 35 years old group and the remaining 232 were included in the 36 years old group. In order to ensure that good prognosis patients were exclusively included in the data set of the current study, the inclusion criterion of an etiology of tubal factor infertility was endorsed. An idiopathic or unexplained infertility factor could present as a confounder, jeopardizing our results, hence a meticulous selection of patients based on their reproductive history was conducted. A flow chart on the study group selection process is presented in Figure 1.

On the day of the hCG triggering, the mean levels of estradiol were 2190.94 ± 116.7 pg/mL, in the range of 1704–2829 pg/mL. In addition, AMH levels were recorded at 2.71–8.36 ng/mL, estimating an average of 5.15 ± 1.40 ng/mL. In regard to FSH and LH mean levels of 6.05 ± 1.20 mIU/mL and 3.91 ± 1.31 mIU/mL were documented, respectively. Minimum and maximum FSH levels were recorded at 3.7–7.8 mIU/mL, while LH levels were in the range of 1.5–6.4 mIU/mL, respectively. A comparison between the three age groups revealed no statistically significant difference in regard to the patients’ hormonal profile.

The accumulated number of oocytes per retrieval was estimated at an average of 14.71 ± 9.21, in the range 10–28. Regarding the number of retrieved oocytes, no statistically significant difference was detected amongst the three age groups (15.76 ± 9.74 vs. 13.89 ± 9.73 vs. 14.24 ± 8.64). No statistically significant difference was noted regarding oocyte fertilization, notably an average of 8.26 ± 5.94 oocytes was documented as a normally fertilized level. Table 1 displays the hormonal levels, the number of retrieved oocytes as well as two-pronuclear (2PN) zygotes documented for each group of patients.

With regard to the frequency of A, B or C quality blastocysts transferred, as one might expect, there was a tendency for younger patients to have better quality blastocysts. However, that difference did not quite reach statistical significance (*p*-value = 0.064), as presented in Table 2.

Concerning a positive hCG rate, a statistically significant difference was observed between the three groups (*p*-value = 0.02). Post-hoc analysis revealed that women aged 36 years old reported a significantly lower positive hCG rate compared to the cases involving women at the age of 34 (*p*-value = 0.009) or 35 (*p*-value = 0.023) years old. Table 3 demonstrates the positive hCG rate for each age group.

In regards to clinical pregnancy rate, women aged 35 reported a higher clinical pregnancy rate compared to women who were 36 years old (*p*-value = 0.05). Moreover, women who were 34 years old also demonstrated a statistically significantly higher clinical pregnancy rate compared to women included in the 36 years old group (*p*-value = 0.04). Table 4 demonstrates the clinical pregnancy rate for each age group.

## 4. Discussion

In the last 30 years, childbearing beyond the age of 35 has become a growing global phenomenon. Consequently, the mean age of first gestation for women is reportedly on the rise. However, this increase in female age implies a firm linear increase [11,12] of reported complications or miscarriages. Therefore, nowadays reversing the adverse effects of AMA in the ART field constitutes a crucial objective for the practitioners. Nonetheless, unexplored territory when determining the optimal treatment regarding women over the age of 35 still remains to be mapped [1,2,24].

Despite an abundance of data presented in narrative reviews regarding a general decline in successful ART procedures with advancing age, there is some lack of concrete data which would allow a robust conclusion to be drawn regarding a specific cut-off point at which critical decisions might be made for management of patients in the AMA category. This discrepancy of evidence is attributed to the fact that the majority of published studies compares cohorts of patients employing "age boxes" described by a large range in age frames between 30 and 35, 35 and 39 or above 40 [3,4,5,8,19,21,25].

Recent literature publications provide discordant verdicts as to whether age 35 or 40 [20,21,22] is the appropriate cut-off point to pursue a certain approach in assisted reproduction. Since age 35 is already considered a cut-off point in the field of gynecology and obstetrics according to guidelines, it is reasonable to ponder on whether this could extend to the implementation of ART, and serve as an age cut-off point in the decision making process for IVF set-up [26]. The present study was designed and executed in an effort to highlight the potential value of adopting age 35 as a cut-off point in clinical practice. Acknowledging the fact that the vast majority of studies investigating age incorporate rather large interval age cohorts in their design, this study uniquely brings to the literature data regarding the associations among groups of patients referring to distinct calendar years 34, 35 and 36. This will contribute in assessing IVF outcomes in cases of good prognosis patients subjected to a first attempt in assisted reproduction.

To delineate the role of age 35 as a cut-off point, a strategy was designed comparing the outcome of IVF between cohorts consisting of 34, 35 and 36 years old patients. The purpose of these strictly defined age groups was to investigate the true impact of actual calendar years on successful fertilization, given the time-sensitivity describing the reproductive age period between 34 and 36 in women requesting services of assisted reproduction. Patients’ data were collected from the Assisted Reproduction Unit. No data on the final outcome of pregnancies, live birth rate or obstetrical history are provided, since a follow-up monitoring of patients often presented with difficulties. The main limitation of this research is its retrospective nature, whereas the final number of patients recruited was dictated by the stringent criteria set to exclude confounders and focus on the real effect of age on IVF response.

Both levels of gonadotropins and the AMH as well as the number of accumulated oocytes showed no statistically significant variations between the three groups. The number of normally fertilized embryos presented with no statistical difference when compared between the groups as well. Proceeding with a subgroup analysis to investigate the quality of transferred embryos amongst groups, the majority of blastocysts were assessed as being top quality in all three age groups, with the absence of a statistical difference between them. Interestingly, concerning positive hCG rate, women at 36 years of age subjected to IVF cycles appear to have a significantly lower positive hCG rate in comparison to the other two age groups. Similarly, women aged 35 presented with significantly higher clinical pregnancy rates following a single IVF cycle, compared to patients included in the age group of 36.

Published studies have come to discrepant conclusions regarding the best protocols for stimulating the ovaries in women over the age of 35, claiming differences in number and quality of accumulated oocytes, implantation and pregnancy rates [27,28,29,30]. The present study demonstrates that irrespectively of the closely defined age-group analysed herein, participants showcased an excellent ovarian response with a standard GnRH agonist long-protocol, showing no significant differences in number of oocytes retrieved between groups. Similarly, no differences in the number of normally fertilized oocytes were recorded either, indicating that maternal age of a single calendar year from 34 to 35 and 36 exerts no impact on fertilization rate.

Defining the right age cut-off point is essential as it can provide answers to many of the questions that divide clinicians’ opinion, particularly when patients transition from one age group to the other. Effective management of issues such as determining the number of embryos to be included for embryo transfer, and opting for the appropriate stimulation protocol is of the utmost importance for achieving optimal results and limiting complications. A strict age cut-off point may contribute towards clarifying the landscape concerning the efficient IVF management for women aged 34 to 36.

The scientific community has yet to conclude on the conundrum on the optimal number of embryos transferred during an IVF cycle. This lack of consensus is heightened to a significant extent by the striking differentiations and inconsistencies in terms of legislation and guidelines established by various countries [16,17]. Thus, a universally accepted consensus regarding the embryo transfer procedure seems rather challenging to concur on. The results of this study may prove of particular value for clinicians during the decision-making process, as a significantly lower positive hCG and clinical pregnancy rates was identified in 36-year-old women compared to in the other two groups. Subsequently, this data could serve as an indication for establishing the age of 35 as the cut-off point in selecting for the optimal number of embryos to be transferred in a single cycle. Aging from 34 to 35 exerts no negative impact on the outcome of fertilization, as opposed to aging over 35 where a single year could exert a significant negative effect and compromise the outcome during an IVF attempt. Thus, it is safe to conclude that the age of 35 years could serve as a valid cut-off point in terms of decision-making concerning the outcome of IVF. In this era of elective single embryo transfer, authors refrain from concluding on the optimal number of embryos transferred for women aged 36. What is more, examining whether including more embryos during the transfer procedure in patients aged 36 could result in enhanced pregnancy rates was not investigated nor considered during design of this study.

The underlying reason behind a compromised implantation and clinical pregnancy potential is considered to be aging. In an effort to shed light into this phenomenon, it has been proposed that placental insufficiency and placenta accrete-both being associated with AMA and the implementation of IVF-could be involved in the pathophysiological mechanism of the age-related decline in the live birth rate [31,32]. In conclusion, the present research focuses on three strictly defined age groups of women presenting with good prognosis IVF patients of good ovarian response upon their first treatment cycle of treatment between 34 and 36 years of age. As stated by the Human Fertilization and Embryology Authority (HFEA), the mean age of women pursuing fertility treatment through IVF was 35.5 years in 2017 compared to the age of 33.5 in 1991 [33]. The age range investigated herein is admittedly considered as the one surrounded by the most controversy when clinicians are deliberating on the optimal number of embryos included in an embryo transfer procedure.

## 5. Conclusions

According to our results, age 35 should reflect a cut-off point determining the number of embryos transferred on the grounds that positive hCG and clinical pregnancy rates are statistically significantly increased in women aged 35 compared to those aged 36 years-old. These results may provide practitioners with valuable insights while concurring on the optimal number of embryos employed, particularly when there is a transition from the cut-off age of 35 to 36. Further research to define cut-off points in ART including large prospective studies and meta-analyses will contribute to the adoption of a common line of approach aiming to mitigate risk and optimize the outcome based on age-associated personalized management of ART.

## Figures and Tables

**Table 1:** Presentation of the average ± standard deviation regarding the hormonal profiling of the patients, the number of retrieved oocytes and the number of normally fertilized oocytes for the three age groups.

	Age 34	Age 35	Age 36
**E2 (pg/mL)**	2179.75 ± 292.25	2199.81 ± 308.42	2194.26 ± 301.88
**AMH (ng/mL)**	5.21 ± 1.36	5.14 ± 1.38	5.08 ± 1.44
**FSH (mIU/mL)**	5.99 ± 1.27	6.01 ± 1.16	6.14 ± 1.20
**LH (mIU/mL)**	3.99 ± 1.27	3.87 ± 1.34	3.88 ± 1.29
**Oocytes Retrieved**	15.76 ± 9.74	13.89 ± 9.73	14.24 ± 8.64
**Two-pronuclear Zygotes**	8.22 ± 5.76	8.39 ± 6.67	8.18 ± 5.38

**Table 2:** Frequency of embryo transfer at day 5 regarding the blastocysts’ quality for each age group.

	Age 34	Age 35	Age 36	*p*-Value
**D5A ^†^**	56.1%	48.2%	46.6%	0.064
**D5B ^‡^**	25.4%	23.1%	26.3%	
**D5C ^§^**	18.5%	28.7%	27.1%	

†: Category D5A consisted of two top quality blastocysts rated as either 4AA, 5AA or 6AA. ‡: Category D5B consisted of a top quality blastocyst along with another non-top quality blastocyst. §: Category D5C consisted of two blastocysts evaluated as non-top quality. Statistical analysis was performed employing a contingency chi-squared test.

**Table 3:** Positive hCG Rate for each age group.

	Age 34	Age 35	Age 36	Age 34 vs. Age 35 (*p*-Value)	Age 34 vs. Age 36 (*p*-Value)	Age 35 vs. Age 36 (*p*-Value)
**Positive**	148 (59.7%)	132 (58.4%)	111 (47.8%)	0.774	0.009	0.023
**Negative**	100 (40.3%)	94 (41.6%)	121 (52.2%)			

Statistical analysis was performed employing a contingency chi-squared test.

**Table 4:** Clinical Pregnancy rate documented for the three distinctive age groups.

	Age 34	Age 35	Age 36	Age 34 vs. Age 35 (*p*-Value)	Age 34 vs. Age 36 (*p*-Value)	Age 35 vs. Age 36 (*p*-Value)
**Pregnant**	134 (54.0%)	122 (54.0%)	104 (44.8%)	0.991	0.04	0.05
**Not Pregnant**	114 (46.0%)	104 (46.0%)	128 (55.2%)			

Statistical analysis was performed employing a contingency chi-squared test.
